# A Prenylated Xanthone, Cudratricusxanthone A, Isolated from *Cudrania tricuspidata* Inhibits Lipopolysaccharide-Induced Neuroinflammation through Inhibition of NF-κB and p38 MAPK Pathways in BV2 Microglia

**DOI:** 10.3390/molecules21091240

**Published:** 2016-09-16

**Authors:** Chi-Su Yoon, Dong-Cheol Kim, Tran Hong Quang, Jungwon Seo, Dae Gill Kang, Ho Sub Lee, Hyuncheol Oh, Youn-Chul Kim

**Affiliations:** 1 Institute of Pharmaceutical Research and Development, College of Pharmacy, Wonkwang University, Iksan 54538, Korea; ycs1991@naver.com (C.-S.Y.); kimman07@hanmail.net (D.-C.K.); sjw315@naver.com (J.S.); hoh@wku.ac.kr (H.O.); 2 Institute of Marine Biochemistry, Vietnam Academy of Science and Technology (VAST), 18 Hoang Quoc Viet, Caugiay, Hanoi 100000, Vietnam; quangth2004@yahoo.com; 3 Hanbang Body-Fluid Research Center, Wonkwang University, Iksan 54538, Korea; dgkang@wku.ac.kr (T.H.Q.); host@wku.ac.kr (H.S.L.)

**Keywords:** *Cudrania tricuspidata*, cudratricusxanthone A, microglia, neuroinflammation, nuclear factor-kappa B, mitogen-activated protein kinase

## Abstract

*Cudrania tricuspidata* Bureau (Moraceae) is an important source of traditional Korean and Chinese medicines used to treat neuritis and inflammation. Cudratricusxanthone A (**1**), a prenylated xanthone, isolated from *C. tricuspidata*, has a variety of biological and therapeutic activities. The goal of this study was to examine the effects of compound **1** on neuroinflammation and characterize its mechanism of action in lipopolysaccharide (LPS)-stimulated BV2 microglia. Cudratricusxanthone A (**1**) suppressed the expression of inducible nitric oxide synthase (iNOS) and cyclooxygenase (COX)-2 enzymes and decreased the production of iNOS-derived nitric oxide and COX-2-derived prostaglandin E2 in LPS-stimulated mouse BV2 microglia. The compound also decreased tumor necrosis factor-α, interleukin (IL)-1β, and IL-12 production; inhibited the phosphorylation and degradation of IκB-α; and blocked the nuclear translocation of p50 and p65 in mouse BV2 microglia induced by LPS. Cudratricusxanthone A (**1**) had inhibitory effects on nuclear factor kappa B DNA-binding activity. Additionally, it inhibited the p38 mitogen-activated protein kinase signaling pathway. Our data suggests that cudratricusxanthone A (**1**) may be a useful therapeutic agent in the treatment of neurodegenerative diseases caused by neuroinflammation.

## 1. Introduction

*Cudrania tricuspidata* (Moraceae) is a deciduous broadleaf thorny tree distributed throughout China, Korea, and Japan. The root of this plant has been used as a Korean and Chinese traditional medicine for the treatment of neuritis and inflammation [1]. *C. tricuspidata* is rich in glycoproteins, xanthones, and flavonoids [2] and has been shown to have various biological effects, including antioxidant effects [3], monoamine oxidase (MAO) A inhibition [4], neuroprotective effects [5], and antiatherosclerotic and anti-inflammatory effects [6]. Our previous phytochemical study of the roots of *C. tricuspidata* yielded nine prenylated xanthones [7], including cudratricusxanthone A (**1**). This compound also has many biological activities, including hepatoprotection [8], anti-inflammation [9], and neuroprotection [10]. During our investigation to identify natural compounds with antineuroinflammatory effects, we focused on cudratricusxanthone A, among the nine prenylated xanthones, to elucidate its antineuroinflammatory effects and mechanism of action.

Microglia, characterized as macrophages-like immune cells, play a critical role in host defense in the central nervous system [11]. Microglia are activated in response to brain damage and release various pro-inflammatory cytokines and mediators, including nitric oxide (NO), reactive oxygen species (ROS), tumor necrosis factor-α (TNF-α), and interleukin (IL)-1β [12,13]. However, aberrant activation of microglia plays a pathogenic role in neuroinflammation, which is the main cause of neurodegenerative diseases [14,15]. Therefore, regulation of microglial activation may be a valuable therapeutic tool for the treatment of neurodegenerative diseases.

Inflammation is a complex processes that leads to arteriosclerosis, inflammatory bowel disease, arthritis, neurodegenerative disorder, septic shock syndrome, and cancer [16,17]. Inflammation is mediated by cytokines and pro-inflammatory genes, including NO synthase (iNOS) and cyclooxygenase-2 (COX-2). Inflammation is initiated by a variety of pathogens through receptor signals that activate the transcription factor nuclear factor kappaB (NF-κB) and mitogen-activated protein kinases (MAPKs).

NF-κB is known to play a vital role in the mediation of immune and inflammatory responses [18]. Under normal conditions, the NF-κB dimers p50 and p65 exist in the cytoplasm in a complex with the inhibitor protein IκB. When IκB is activated, it is phosphorylated and then ubiquitinated, leading to its degradation. Subsequently, the free NF-κB dimer translocates to the nucleus and binds to the kappaB (κB) sites, promoting the transcription of various pro-inflammatory enzymes [19,20,21].

MAPKs are a major family of kinases associated with the inflammation process. MAPKs have crucial roles in the activation of NF-κB [18,22]. Moreover, lipopolysaccharide (LPS) can activate NF-κB and MAPKs, including extracellular signal-regulated kinase (ERK), c-Jun N-terminal kinase (JNK), and p38 MAPK. These proteins then modulate cytokine production and the expression of pro-inflammatory enzymes, such as NF-κB, iNOS, COX-2, TNF-α, and IL-1β [23,24]. Therefore, NF-κB and MAPK are crucial elements in the inflammatory process and essential targets for anti-inflammatory molecules. In this study, we examined the mechanisms through which cudratricusxanthone A exerts anti-inflammatory effects in LPS-stimulated BV2 microglia.

## 2. Results

### 2.1. Structures of Prenylated Xanthones ***1***–***9***

**Figure 1:**
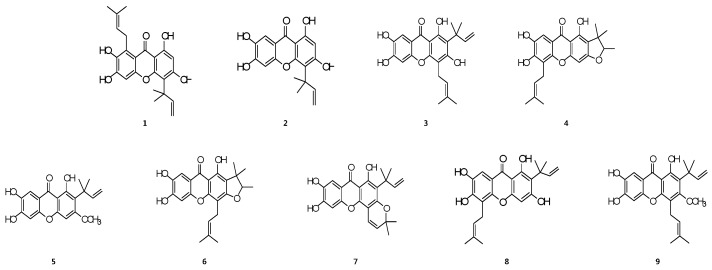
Chemical structures of compounds **1**–**9**.

The structures of cudratricusxanthone A (**1**), cudratricusxanthone L (**2**), cudracuspixanthone A (**3**), cudraxanthone M (**4**), 1,6,7-trihydroxy-2-(1,1-dimethyl-2-propenyl)-3-methoxyxanthone (**5**), cudraxanthone D (**6**), cudratricusxanthone N (**7**), cudraxanthone L (**8**), and macluraxanthone B (**9**) (Figure 1) were determined in a previous study [8].

### 2.2. Effects of Compounds ***1***–***9*** on the Production of Nitric Oxide in LPS-Stimulated BV2 Microglia

**Figure 2:**
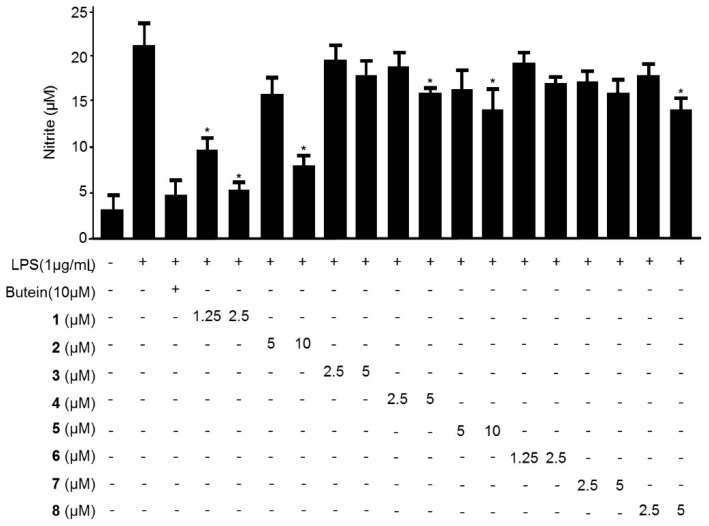
The effects of compounds **1**–**9** on NO production in BV2 microglia stimulated with LPS. The cells were pretreated for 3 h with the indicated concentrations of compounds **1**–**9** and stimulated for 24 h with LPS (1 μg/mL). The LPS treatment was performed in the presence of compound. The concentrations of nitrite were determined as described in the Experimental Section. The data show the means ± SDs of three experiments. * *p* < 0.05 compared with the group treated with LPS. “+” is treated, “−“ is not treated.

To evaluate the antineuroinflammatory effects of compounds **1**–**9** on LPS-stimulated BV2 microglia, the concentrations of the pro-inflammatory mediator NO were assessed in the presence and absence of compounds **1**–**9** at noncytotoxic concentrations (data not shown). The next experiments were conducted at the noncytotoxic concentration. BV2 microglia were pretreated with compounds **1**–**9** for 3 h, followed by stimulation with LPS (1 µg/mL) for 24 h. As shown in Figure 2, LPS treatment triggered an approximately 8-fold increase in nitrite concentration in the culture medium compared with that of the untreated group. However, pre-treatment of the cells with compounds **1**–**9** for 3 h decreased the production of NO as indicated by the nitrite concentration in a concentration-dependent manner, with an IC_50_ values of 0.98 ± 0.05, 7.47 ± 0.37, 11.30 ± 0.57, 10.66 ± 0.53, 13.77 ± 0.69, 20.65 ± 1.03, 19.44 ± 0.97, 11.19 ± 0.56, 6.27 ± 0.31 μM, respectively. Cudratricusxanthone A (**1**) was the most potent inhibitor of LPS-induced NO production. This observation led us to further investigate the effects of compound **1** on the production of the LPS-induced pro-inflammatory cytokines TNF-α, IL-1β, IL-12, and IL-6.

### 2.3. Effects of Cudratricusxanthone A *(**1**)* on the mRNA Expression of the Pro-Inflammatory Cytokines TNF-α, IL-1β, IL-12, and IL-6 in LPS-Stimulated BV2 Microglia

**Figure 3:**
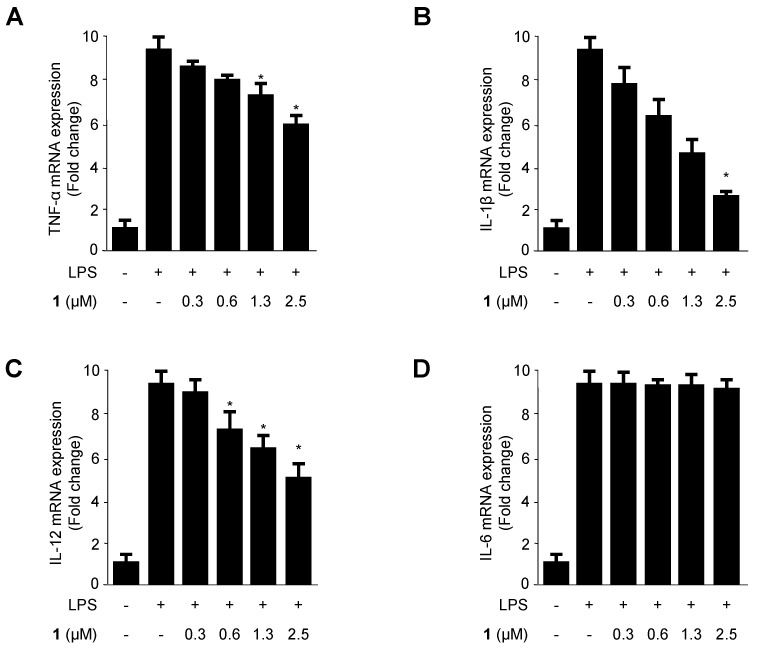
The effects of cudratricusxanthone A (**1**) on TNF-α (**A**); IL-1β (**B**); IL-12 (**C**); and IL-6 (**D**) mRNA expression in LPS-stimulated BV2 cells. Cells were pretreated for 3 h with the indicated concentrations of cudratricusxanthone A (**1**) and then stimulated for 12 h with LPS (1 μg/mL). The LPS treatment was performed in the presence of compound. The concentrations of TNF-α (**A**), IL-1β (**B**); IL-12 (**C**); and IL-6 (**D**) were determined as described in the Experimental Section. RNA quantification was performed as described in the Experimental Section, and representative blots of three independent experiments are shown. The data represent the means ± SDs of three experiments. * *p* < 0.05 compared with the group treated with LPS. “+” is treated, “−“ is not treated.

Next, we investigated the effects of cudratricusxanthone A (**1**) on the production of pro-inflammatory cytokines (TNF-α, IL-1β, IL-12, and IL-6) in BV2 microglia. Cells were pretreated with compound **1** at different concentrations for 3 h and then treated with LPS. As shown in Figure 3A–C, cudratricusxanthone A decreased TNF-α, IL-1β, and IL-12 production in a concentration-dependent manner, with TNF-α IC_50_ value of >2.5 μM, IL-1β IC_50_ value of 1.02 ± 0.05 μM, IL-12 IC_50_ value of 2.22 ± 0.11 μM, as measured by qRT-PCR . However, the production of IL-6 was not altered.

### 2.4. Effects of Cudratricusxanthone A *(**1**)* on PGE_2_ Production and iNOS and COX-2 Protein Expression in LPS-Stimulated BV2 Microglia

**Figure 4:**
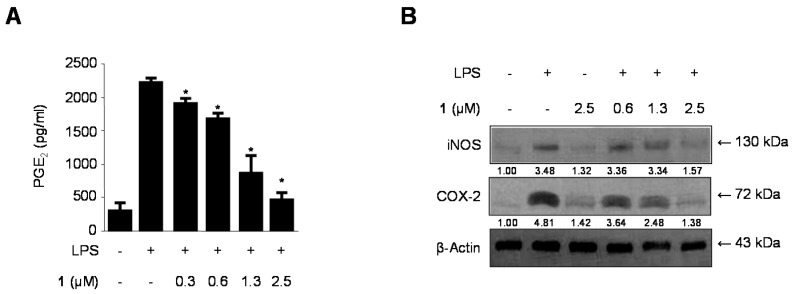
The effects of cudratricusxanthone A (**1**) on the PGE_2_ production (**A**) and protein expression of iNOS and COX-2 (**B**) in BV2 microglia stimulated with LPS. Cells were pretreated for 3 h with the indicated concentrations of cudratricusxanthone A (**1**) and then stimulated for 24 h with LPS (1 μg/mL). The LPS treatment was performed in the presence of compound. The concentrations of iNOS and COX-2 (**B**) were determined as described in the Experimental Section. Western blot analyses were performed as described in the Experimental Section, and representative blots of three independent experiments are shown. The band intensity was quantified by densitometry and normalized to β-actin, and the values are presented at the bottom of the each band. Relative data represent the means ± SDs of three experiments. * *p* < 0.05 compared with the LPS-treated group. “+” is treated, “−“ is not treated.

We next investigated the effects of cudratricusxanthone A (**1**) on LPS-induced PGE_2_ production and iNOS and COX-2 protein expression (Figure 4). BV2 microglia were challenged with LPS (1 μg/mL) in the presence or absence of compound **1** at noncytotoxic concentrations ranging from 0.3 to 2.5 µM. Pretreatment of the microglia with cudratricusxanthone A (**1**) for 3 h resulted in a decrease in iNOS expression (Figure 4B) and reduction of COX-2-derived PGE_2_ (Figure 4A) production, with IC_50_ value of 0.84 ± 0.04 μM. Under the same conditions, compound **1** also suppressed COX-2 expression (Figure 4B).

### 2.5. Effects of Cudratricusxanthone A *(**1**)* on IκB-α Levels, NF-κB Nuclear Translocation, and NF-κB DNA Binding Activity in LPS-Stimulated BV2 Microglia

The activation of NF-κB is essential for the expression of iNOS and COX-2 genes. Under normal conditions, NF-κB is inactive in the cytoplasm because it is bound to its inhibitor, IκB. In response to external signals, NF-κB is released from IκB and subsequently translocates to the nucleus [20,25].

**Figure 5:**
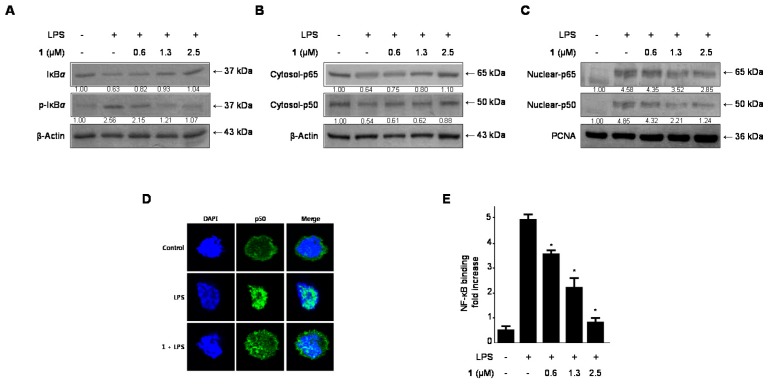
The effects of cudratricusxanthone A (**1**) on IκB-α phosphorylation and degradation (**A**); NF-κB translocation (**B**,**C**); NF-κB localization as determined by immunofluorescence analysis (**D**); and NF-κB DNA binding activity (**E**) in BV2 microglia. Cells were pretreated for 3 h with the indicated concentrations of cudratricusxanthone A (**1**), and stimulated for 1 h with LPS (1 μg/mL). The LPS treatment was performed in the presence of compound. Western blot analyses of IκB-α and *p*-IκB-α in the cytoplasm (**A**) and NF-κB in the cytoplasm (**B**) and nucleus (**C**) and immunofluorescence analyses (**E**) were carried out as described in the Experimental Section. The band intensity was quantified by densitometry and normalized to β-actin and PCNA, and the values are presented at the bottom of the each band. Relative data represent the means ± SDs of three experiments. * *p* < 0.05 compared with the LPS-treated group. “+” is treated, “−“ is not treated.

Therefore, we next examined whether cudratricusxanthone A (**1**) inhibited IκB-α phosphorylation and degradation, thus blocking NF-κB (p50 and p65) nuclear translocation. As shown in Figure 5A, IκB-α was degraded after exposure of BV2 microglia to LPS for 1 h. However, 3 h pretreatment with compound **1** (0.6 to 2.5 μM) markedly suppressed this LPS-induced phosphorylation and degradation of IκB-α in a concentration-dependent manner, inhibiting p50 and p65 translocation to the nucleus (Figure 5B,C). Furthermore, we confirmed this phenomenon using fluorescence microscopy, which showed that NF-κB nuclear translocation was decreased in treated cells compared with that in untreated microglia (Figure 5D). We also investigated the DNA binding activity of NF-κB in nuclear extracts of BV2 microglia stimulated with LPS for 1 h. This treatment induced an approximate 10-fold increase in NF-κB DNA binding activity, which was inhibited by cudratricusxanthone A (**1**) in a concentration-dependent manner, with IC_50_ value of 0.92 ± 0.46 μM (Figure 5E).

### 2.6. Effects of Cudratricusxanthone A *(**1**)* on the Phosphorylation of MAPKs in BV2 Microglia Stimulated with LPS

**Figure 6:**
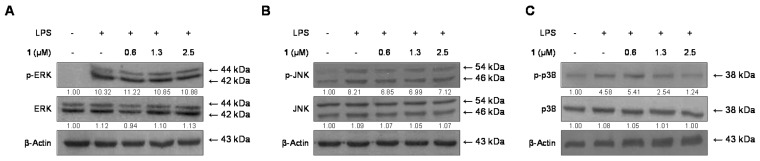
Effects of cudratricusxanthone A (**1**) on ERK, JNK, and p38 MAPK phosphorylation and protein expression. Cells were pretreated for 3 h with the indicated concentrations of cudratricusxanthone A (**1**) and stimulated for 1 h with LPS (1 μg/mL). The LPS treatment was performed in the presence of compound. The levels of (**A**) phosphorylated ERK (p-ERK); (**B**) phosphorylated JNK (p-JNK); and (**C**) phosphorylated p38 MAPK (p-p38 MAPK) were determined by western blotting. Representative blots from three independent experiments with similar results and densitometric evaluations are shown. Band intensity was quantified by densitometry and normalized to β-actin, and the values are presented at the bottom of each band. “+” is treated, “−“ is not treated.

To investigate the MAPK-mediated suppression of inflammatory reactions by cudratricusxanthone A (**1**), we assessed its effects on the LPS-induced phosphorylation of ERK, JNK, and p38 in BV2 microglia cells. As shown in Figure 6, the phosphorylation levels of ERK, JNK, and p38 increased after treatment with LPS for 1 h. However, pretreatment with cudratricusxanthone A (**1**) (0.6–2.5 μM) for 3 h, significantly inhibited the LPS-induced phosphorylation of p38 in a concentration-dependent manner (Figure 6C). ERK and JNK phosphorylation were not affected. Notably, the expression levels of ERK, JNK, and p38 were also not affected by LPS. These data suggested that compound **1** regulated inflammatory reactions by inhibiting the p38 MAPK signaling pathway.

## 3. Discussion

Cudratricusxanthone A (**1**), a prenylated xanthone isolated from *C. tricuspidata*, has been reported to have diverse biological effects, including hepatoprotective [8,26], neuroprotective [10], antiplatelet, anticoagulant, profobrinolytic [27], pancreatic beta cell-protective [28], and human cytochrome P450 inhibitory activities [29]. Additionally, although this compound has been shown to have anti-inflammatory effects in LPS-induced RAW264.7 cells [8], the antineuroinflammatory mechanisms of cudratricusxanthone A had not yet been examined in BV2 microglial cells.

Microglia are brain resident macrophages present in the central nervous system (CNS) [30,31]. Microglia play a pivotal role in the pathogenesis of various neurologic disorders, such as Huntington’s disease, Alzheimer’s disease, Parkinson’s disease, and amyotrophic lateral sclerosis [32,33]. The activation of microglia cells generates several cellular responses that play crucial roles in the pathogenesis of inflammation [34,35]. A variety of pro-inflammatory cytokines and neurotoxic mediators produced by activated microglia are known to contribute to neuronal injury and the pathogenesis of neuroinflammatory diseases. Therefore, the regulation of pro-inflammatory mediator production could be a promising target for neuroinflammation-related diseases [36,37]. Recently, the concept of multiple phenotypes for microglia has obtained much interest: Stimulation of the M1 phenotype processes neuroinflammation while activation of the M2 phenotype raises the production of anti-inflammatory factors [38,39]. Therefore, the anti-neuroinflammatory effects of CTXA could be linked to its preventive effect on microglia activation through polarization to an anti-inflammatory M2 phenotype. However, further studies are required to clarify the effects of CTXA on M1/M2 phenotypes of microglia.

Recent studies have shown that inflammation is generated by various pro-inflammatory cytokines and mediators, such as PGE_2_, TNF-α, IL-6, IL-12, and IL-1β in immune cells [21,40,41]. NO is a free radical known to act as an inflammatory mediator in the process of microglia-mediated inflammation in the CNS [42]. NO production is catalyzed by iNOS, and regulation of NO is an excellent strategy for treating neuroinflammatory diseases [43,44]. Therefore, we examined whether prenylated xanthone derivatives suppressed the production of NO in LPS-induced BV2 microglia. Of the compounds examined, cudratricusxanthone A (**1**) showed the most potent effects on the inhibition of NO production among the nine tested compounds (Figure 2). PGE_2_, an inflammatory mediator, is a by-product of COX-2 that induces the development of inflammatory diseases [35]. First, we examined whether iNOS and COX-2 could be target enzymes for the anti-inflammatory mechanism. We determined whether cudratricusxanthone A (**1**) inhibited iNOS and COX-2 expression in LPS-induced BV2 microglia. Our results showed that LPS induced iNOS and COX-2 protein expression in BV2 microglia and that these changes could be inhibited by pretreatment with cudratricusxanthone A (**1**) (Figure 4B). Compound **1** also repressed the COX-2-dependent generation of PGE_2_ and downregulated pro-inflammatory cytokines, such as TNF-α, IL-1β, and IL-12 (Figure 3A–C and Figure 4A).

The transcription factor NF-κB is an important molecule in several pathologic conditions and has been shown to mediate inflammatory responses [18]. NF-κB, originally designated the p50–p65 heterodimer, exists in its inactive form in a complex with its inhibitory protein (IκB) in the cytoplasm under normal conditions. Upon stimulation by a variety of stimuli, such as LPS and IL-1β, the IκB protein is phosphorylated and degraded, resulting in translocation of free NF-κB (p50 and p65) to the nucleus [20]. In the nucleus, NF-κB binds to DNA binding sites and promotes the transcription of various pro-inflammatory mediators and cytokines, such as NO, PGE_2_, TNF-α, IL-6, IL-12, and IL-1β [21,35]. Therefore, we examined the effects of cudratricusxanthone A (**1**) on IκB-α phosphorylation and degradation and on NF-κB heterodimer (p65 and p50) translocation. Following treatment with cudratricusxanthone A (**1**), LPS-induced IκBα degradation and NF-κB activation were inhibited in BV2 microglia cells (Figure 5A–C). In addition, cudratricusxanthone A (**1**) decreased the DNA binding activity of NF-κB (Figure 5E).

MAPKs, including p38, JNK, and ERK1/2 (p44/p42), are an important kinase family involved in various cellular processes, such as differentiation, stress responses, apoptosis, and immune defense [22]. Furthermore, MAPKs have critical roles in inducing cytokine production [23]. Thus, NF-κB and MAPK are crucial molecules in the inflammatory process and are important targets of anti-inflammatory molecules. Accordingly, further experiments were conducted to determine whether compound **1** regulated the expression of MAPKs to induce anti-inflammatory effects in LPS-stimulated microglia. Our results demonstrated that cudratricusxanthone A (**1**) was a potent inhibitor of LPS-induced p38 activation in BV2 microglia (Figure 6C), suggesting that the anti-inflammatory effects of cudratricusxanthone A (**1**) were mediated by suppression of the MAPK signaling pathway.

In summary, some prenylated xanthone derivatives exert anti-neuroinflammatory effects through suppression of NO production. In particular, in this study, we showed that cudratricusxanthone A (**1**) exerted its anti-neuroinflammatory effects by inhibiting the expression of various pro-inflammatory mediators and the activation of NF-κB and p38 MAPK in LPS-stimulated BV2 microglial cells. Thus, mediating the production of inflammatory molecules by cudratricusxanthone A (**1**) may have therapeutic potential for the treatment of various neurodegenerative diseases.

## 4. Experimental Section

### 4.1. Chemicals and Reagents

The nine prenylated xanthone derivatives were obtained in our previous study [8]. Dulbecco’s modified Eagle’s medium (DMEM), fetal bovine serum (FBS), and other tissue culture reagents were purchased from Gibco BRL Co. (Grand Island, NY, USA). All other chemicals were obtained from Sigma Chemical Co. (St. Louis, MO, USA). Primary antibodies, including mouse/goat/rabbit anti-COX-2, anti-iNOS, anti-β-actin, anti-IкB-α, anti-phospho-IкB-α, anti-p50, anti-p65, and anti-PCNA, and secondary antibodies were purchased from Santa Cruz Biotechnology (Heidelberg, Germany). Anti-phospho-ERK, anti-ERK, anti-phospho-JNK, anti-JNK, anti-phospho-p38, and anti-p38 antibodies were obtained from Cell Signaling Technology (Danvers, MA, USA).

### 4.2. Cell Culture and Viability Assay

BV2 microglia cells were a gift from Prof. Hyun Park at Wonkwang University (Iksan, Korea). BV2 cells were maintained at 5 × 10^6^ cells/dish in 100-mm dishes in DMEM supplemented with 10% heat-inactivated FBS, penicillin G (100 U/mL), streptomycin (100 mg/mL), and l-glutamine (2 mM) and incubated at 37 °C in a humidified atmosphere containing 5% CO_2_ and 95% air. For determination of cell viability, cells (2 × 10^4^ cells/well in 96-well plates) were incubated with 3-(4,5-dimethylthiazol-2-yl)-2,5-diphenyltetrazolium bromide (MTT) at a final concentration of 0.5 mg/mL for 3 h, and the formazan formed was dissolved in acidic 2-propanol. The optical density was measured at 590 nm with a microplate reader (BioRad, Hercules, CA, USA). The optical density of the formazan formed in control (untreated) cells was considered to represent 100% viability [45,46].

### 4.3. Quantitative Reverse Transcription Polymerase Chain Reaction (PCR)

Total RNA was isolated from the cells using TRIzol (Invitrogen, CA, USA), in accordance with the manufacturer’s recommendations, and quantified spectrophotometrically at 260 nm. Total RNA (1 μg) was reverse transcribed using a High Capacity RNA-to-cDNA kit (Applied Biosystems, Carlsbad, CA, USA). The cDNA was then amplified using a SYBR Premix Ex Taq kit (TaKaRa Bio, Shiga, Japan) with a StepOnePlus Real-Time PCR system (Applied Biosystems, Foster City, CA, USA). Briefly, reactions were carried out in a 20 μL reaction volume containing 10 μL of SYBR Green PCR Master Mix, 0.8 μM of each primer, and diethyl pyrocarbonate (DEPC)-treated water. The primer sequences were designed using Primer Quest (Integrated DNA Technologies, Cambridge, MA, USA). The primer sequences were as follows: forward and reverse primers for TNF-α, 5′-CCAGACCCTCACACTCACAA-3′ and 5′-ACAAGGTACAACCCATCGGC-3′; forward and reverse primers for IL-1β, 5′-AATTGGTCATAGCCCGCACT-3′ and 5′-AAGCAATGTGCTG GTGCTTC-3; forward and reverse primers for IL-6, 5′-ACTTCACAAGTCGGAGGCTT-3′ and 5′-TGCAAGTGCATCATCGTTGT-3′; and forward and reverse primers for IL-12, 5′-AGTGACATGTG GAATGGCGT-3′ and 5′-CAGTTCGGGCAGGGTCT-3′. The optimum conditions for PCR amplification of the cDNA were established by following the manufacturer's instructions. The data were analyzed using Step One software (Applied Biosystems), and the cycle number at the linear amplification threshold (Ct) values for the endogenous control gene (*GAPDH*) and the target gene were recorded [46].

### 4.4. DNA Binding Activity of NF-κB

Microglia were pretreated for 3 h with the indicated concentrations of cudratricusxanthone A (**1**) and then stimulated for 1 h with LPS (1 μg/mL). The DNA-binding activity of NF-κB in nuclear extracts was measured using a TransAM kit (Active Motif, Carlsbad, CA, USA) according to the manufacturer’s instructions [45].

### 4.5. Preparation of Cytosolic and Nuclear Fractions

BV2 microglial cells were homogenized in PER-Mammalian Protein Extraction Buffer (1:20, *w*/*v*; Pierce Biotechnology, Rockford, IL, USA) containing freshly added protease inhibitor cocktail I (EMD Biosciences, San Diego, CA, USA) and 1 mM phenylmethylsulfonly fluoride (PMSF). The cytosolic fraction of the cells was prepared by centrifugation at 16,000× *g* for 5 min at 4 °C. The nuclear and cytoplasmic cell extracts were prepared with NE-PER nuclear and cytoplasmic extraction reagents (Pierce Biotechnology), respectively [45].

### 4.6. Nitrite (NO Production) Determination

The nitrite concentration in the medium, an indicator of NO production, was measured with the Griess reaction. Each supernatant (100 µL) was mixed with an equal volume of the Griess reagent (Solution A: 222488, Solution B: S438081; Sigma), and the absorbance of the mixture at 525 nm was determined using an enzyme-linked immunosorbent assay (ELISA) plate reader [45].

### 4.7. Western Blot Analysis

BV2 microglial cells were harvested and pelleted by centrifugation at 16,000 rpm for 15 min. The cells were then washed with PBS and lysed with 20 mM Tris-HCl buffer (pH 7.4) containing a protease inhibitor mixture (0.1 mM PMSF, 5 mg/mL aprotinin, 5 mg/mL pepstatin A, and 1 mg/mL chymostatin). The protein concentration was determined with a Lowry protein assay kit (P5626; Sigma). An equal amount of protein from each sample was resolved using sodium dodecyl sulfate polyacrylamide gel electrophoresis (SDS-PAGE) on 7.5% and 12% gels and then electrophoretically transferred to Hybond enhanced chemiluminescence (ECL) nitrocellulose membranes (BioRad). The membranes were blocked with 5% skimmed milk and sequentially incubated with the primary antibody (Santa Cruz Biotechnology) and horseradish peroxidase-conjugated secondary antibody followed by ECL detection (Amersham Pharmacia Biotech, Piscataway, NJ, USA) [46].

### 4.8. NF-κB Localization and Immunofluorescence

To study NF-κB localization, cells were grown on Lab-Tek II chamber slides and treated with 2.5 μM cudratricusxanthone A (**1**) for 60 min. Cells were then fixed in formalin and permeabilized with cold acetone. The cells were then probed with anti-p50 antibodies, followed by FITC-labeled secondary antibodies (Alexa Fluor 488; Invitrogen). To visualize the nuclei, cells were then treated with 1 µg/mL DAPI for 30 min, washed with phosphate-buffered saline (PBS) for 5 min, and treated with 50 μL of VectaShield (Vector Laboratories, Burlingame, CA, USA). Stained cells were visualized using a Zeiss fluorescence microscope and photographed (Provis AX70, Olympus Optical Co., Tokyo, Japan) [47].

### 4.9. Statistical Analysis

The data are expressed as the mean ± standard deviation (SD) of at least three independent experiments. To compare three groups, one-way analysis of variance was used, followed by Tukey’s multiple comparison tests. Statistical analyses were performed with GraphPad Prism software, version 3.03 (GraphPad Software Inc., San Diego, CA, USA) [47].

## 5. Conclusions

In summary, our results demonstrated that cudratricusxanthone A (**1**) exerted anti-inflammatory effects by suppressing LPS-induced production of pro-inflammatory mediators through inhibition of NF-κB and MAPK pathways in BV2 microglial cells. Therefore, compound **1** may be a potential chemotherapeutic candidate for the management of neurodegenerative disorders.
